# The Deciduous Teeth—Their Uses and Diseases

**Published:** 1899-10

**Authors:** J. W. Juett

**Affiliations:** Emineoce, Ky.


ARTICLE VIII.

### THE DECIDUOUS TEETH—THEIR USES AND DISEASES.

DR. J. W. JUETT. D. D. S., EMINEOCE, KY.

Read before the Kentucky State Dental Association at Mammoth Cave, May 16, 1899.

I believe the care of children's teeth gives the dentist more trouble and causes him more anxiety than all the rest of his practice with one exception. What is that, say you? If he be a country dentist I would say, making artificial dentures.

I am inclined to think the trouble would be somewhat mitigated and the anxiety reduced if parents were taught the importance of the deciduous teeth.

In all nature I know of nothing more wonderful than the growth and development of the deciduous teeth, their absorption and replacement by the permanent teeth. If you have given the subject much thought or study you know how very important it is to preserve the deciduous teeth and not neglect them because the child will lose them. First, the roots of the deciduous teeth will not be absorbed if the pulp be dead; second, the permanent teeth will not be nourished properly if absorption does not take place in the deciduous teeth; fourth, if the deciduous teeth are left in the mouth with dead pulps they cause the permanent teeth to deflect in or out as the case may be, forming an irregularity; fourth, if the deciduous teeth be removed you leave a cicatrix, which will give you the same defect (probably) that would occur if they were left in the mouth; fifth, the fact of the child's having a mouth full of decayed teeth undoubtedly causes an unhealthy condition there, and not being able to masticate the food properly would give rise to unhealthy conditions of the stomach and a general disarrangement of the alimentary canal; sixth, when the deciduous teeth are well taken care of the permanent teeth in ninety-nine cases in a hundred will be well formed and seldom irregular.

Now, I do not deem it necessary to take up the several divisions of the subject and discuss them, because they have been brought before your minds and you are well acquainted with the facts as stated. The question is, how will we get parents to know the importance and acquaint them with the facts concerning the deciduous teeth? So many mothers bring their children to us with the sixth-year molar giving pain, and when we tell them it is a permanent tooth that is giving the trouble they are very much surprised and generally exclaim, "Well, I thought she would 'shed' that tooth." (Yes, like a snake sheds his skin, maybe yearly!), and if we try to explain the situation to the mother it is hard for her to understand why the tooth must not come out. Perhaps the child is too young to have the nerve removed and we are compelled to remove the tooth. In nine cases out of ten we make an enemy of the child and are fortunate indeed if we can regain its confidence for future operations. This is only one of the many problems that present themselves to us day by day.

Several ways suggest themselves to my mind as to how we can best inform parents concerning this subject.

First, I would say the physician could do much if he were trained, but, however, very few of our medical friends could tell a temporary tooth from a permanent one. I would, therefore, suggest a chair be added to the curriculum of the medical schools and the M D. be required to pass an examination on the formation and care of the teeth so that he could intelligently aid his brother dentist instead of oftentimes hindering him. I have often seen good teeth sacrified because the patient would first visit a physician instead of consulting a dentist.

Then, too, the dentist with such literature as can now be procured should endeavor to educate as much as he can through his patients, and I think he should be progressive enough to reach the teacher and pupils in our public schools with a series of lessons on the care of the teeth. You will be surprised to find how readily the trustees and teachers will give you their assistance.

I present you the models of the mouth of a child eight years of age. I have had the care of her teeth since she was three, putting in the first fillings at that age. She has never had an ache or a pain with her teeth and every tooth that has decayed has been filled. I found some of her teeth quite sensitive, and when I could not excavate sufficiently I would place a small portion of silver nitrate in the tooth and after a few days excavate and fill. You will find this a very great aid in the treatment of children's teeth. I realize that it sometimes tries one's patience and endurance, but I think the time well spent, and it is remarkable how much you can do for the child if you have gained its confidence. I realize, hewever, that some dentists have very little patience of this kind, and I would not advise them to undertake very much.—*Indiana Dental Journal.*

